# Estimation of Arterial Viscosity Based on an Oscillometric Method and Its Application in Evaluating the Vascular Endothelial Function

**DOI:** 10.1038/s41598-019-38776-4

**Published:** 2019-02-22

**Authors:** Hiroshi Tanaka, Akihisa Mito, Harutoyo Hirano, Zu Soh, Ryuji Nakamura, Noboru Saeki, Masashi Kawamoto, Yukihito Higashi, Masao Yoshizumi, Toshio Tsuji

**Affiliations:** 1 Department of System Cybernetics, Graduate School of Engineering, Hiroshima University, Higashi-Hiroshima, 739-8527 Japan; 2 Academic Institute, College of Engineering, Shizuoka University, Hamamatsu, 432-8561 Japan; 3 Department of System Cybernetics, Faculty of Engineering, Hiroshima University, Higashi-Hiroshima, 739-8527 Japan; 4 Department of Anesthesiology and Critical Care, Graduate School of Biomedical and Health Sciences, Hiroshima University, Hiroshima, 734-8553 Japan; 5 Department of Regeneration and Medicine, Research Center for Radiation Genome Medicine, Research Institute for Radiation Biology and Medicine, Hiroshima University, Hiroshima, 734-8553 Japan; 6 Division of Regeneration and Medicine, Hiroshima University Hospital, Hiroshima, 734-8551 Japan; 7 Department of Cardiovascular Physiology and Medicine, Graduate School of Biomedical and Health Sciences, Hiroshima University, Hiroshima, 734-8553 Japan

## Abstract

This paper proposes an algorithm for estimating the arterial viscosity using cuff pressures and pulse waves measured by an automatic oscillometric sphygmomanometer. A change in the arterial viscosity during the enclosed-zone flow-mediated dilation test is calculated as an index for evaluating the vascular endothelial function %*η*. In all, 43 individuals participated in this study. After the index %*η* was calculated, the accuracy of the index %*η* in distinguishing healthy subjects and subjects at a high risk of arteriosclerosis was tested via a receiving operating characteristic (ROC) analysis. The calculated %*η* for the healthy participants and those at a high risk of arteriosclerosis was 13.4 ± 55.1% and −32.7 ± 34.0% (mean ± S.D.), respectively. The area under the ROC curve was 0.77. Thus, it was concluded that the proposed method can be used to evaluate the vascular endothelial function.

## Introduction

In recent years, cardiovascular diseases such as heart diseases and cerebrovascular diseases have accounted for approximately 25% of the deaths among the Japanese people^1^. One of the main causes of these cardiovascular diseases is arteriosclerosis, which must be diagnosed and treated early as it is a progressive and refractory disease. Vascular endothelial dysfunction is known as an early symptom of arteriosclerosis^2^. The vascular endothelial function associated with the sympathetic adrenergic system is involved in fine tuning of the blood pressure. Specifically, the activation of the *α*_1_ adrenergic receptor and *β* adrenergic receptor causes vasoconstriction and nitric oxide (NO)-mediated vasodilatation, respectively^3,4^. The vascular endothelial function can thus be evaluated via changes in the blood flow rate, vascular diameter or arterial mechanical properties, which are associated with vasoconstriction and vasodilatation.

Evaluation methods for vascular endothelial function have been extensively studied since the 1980s, and the first attempt involved invasive methods^5–9^. For example, Panza *et al*. used a plethysmograph to estimate changes in the blood flow rate when a NO agonist or antagonist was administered in an artery^10^. The flow-mediated dilation (FMD) test^11^ was then proposed as a clinically practical method that enabled noninvasive evaluation of the vascular endothelial function. In the FMD test, shear stress is applied on the endothelium to induce NO release, and the dilation ratio of the vascular diameter between the pre- and post-cuff occlusion (*%FMD*) is measured as an index to evaluate the endothelial function. One of the drawbacks of the FMD test is that it requires dexterity because the prolonged stable measurement of the vascular diameter with an ultrasound device is often difficult. To simplify the measurement procedure, a technique applying an oscillometric method^12^, which is widely used in commercial automatic sphygmomanometers, was proposed^13,14^. This technique is referred to as the enclosed-zone flow-mediated dilation (ezFMD) test. In the ezFMD test, a cuff is attached to the upper arm and the cuff pulse wave is measured. The vascular endothelial function is then estimated based on the maximum amplitude change rate between the pre- and post-cuff occlusion of the cuff pulse wave (*%ezFMD*).

In addition, evaluation methods focusing on the arterial mechanical characteristics have been attracted attention since the 2000s^5–9^ because it is a rather direct measure of vasodilation compared to the vascular diameter, which can be affected by changes in the blood pressure. In these studies, several measures such as the pulse wave velocity were used to evaluate the arterial stiffness or viscoelasticity. Our research group has previously proposed a log-linearized peripheral arterial model^15^, which enabled the estimation of the changes in the arterial viscoelasticity in a beat-to-beat manner during the FMD test^16^. These recent studies revealed the effectiveness of the use of viscoelasticity in vascular endothelial function evaluation; however, such methods inherit the drawbacks of the FMD test because viscoelasticity estimation in these cases is carried out based on the vascular diameters measured using the ultrasonic devices.

To improve the clinical applicability, this paper presents a concept employing the oscillometric method and an algorithm for estimating the arterial viscosity to assess the vascular endothelial function. The proposed method enables the estimation of the arterial viscosity from only a common oscillometric automatic sphygmomanometer. The algorithm is then tested in terms of its ability of evaluating the vascular endothelial function by measuring the changes in the estimated viscosity induced by reactive hyperaemia.

## Results

### Baseline clinical characteristics

The baseline clinical characteristics of the participants are summarised in Table 1. The age range was 19–84 years. The mean value of the systolic blood pressure was 126.9 ± 19.2 mmHg, and that of the diastolic blood pressure was 68.0 ± 13.8 mmHg.

**Table 1:** Clinical characteristics of the participants.

Variables	Total (*n* = 43)	Healthy group (*n* = 29)	High-risk group (*n* = 14)	*p*-value
Age,years	34.9 ± 20.9	21.1 ± 1.85	63.6 ± 9.24	1.59 × 10^−10^
Systolic blood pressure, mmHg	126.9 ± 19.2	123.6 ± 15.6	133.9 ± 24.3	0.16
Diastolic blood pressure, mmHg	68.0 ± 13.8	66.8 ± 13.1	70.5 ± 15.2	0.44
Flow-mediated vasodilation, %	6.24 ± 2.30	6.60 ± 2.38	5.49 ± 1.99	0.12
Change ratio of arterial viscosity, %	2.33 ± 53.9	17.6 ± 53.8	−29.3 ± 39.5	2.78 × 10^−3^

All results are presented as mean ± S.D.

*p*-value shows the result of Welch’s *t* test between normal and risk values.

### Viscosity estimation experiment

**Figure 1:**
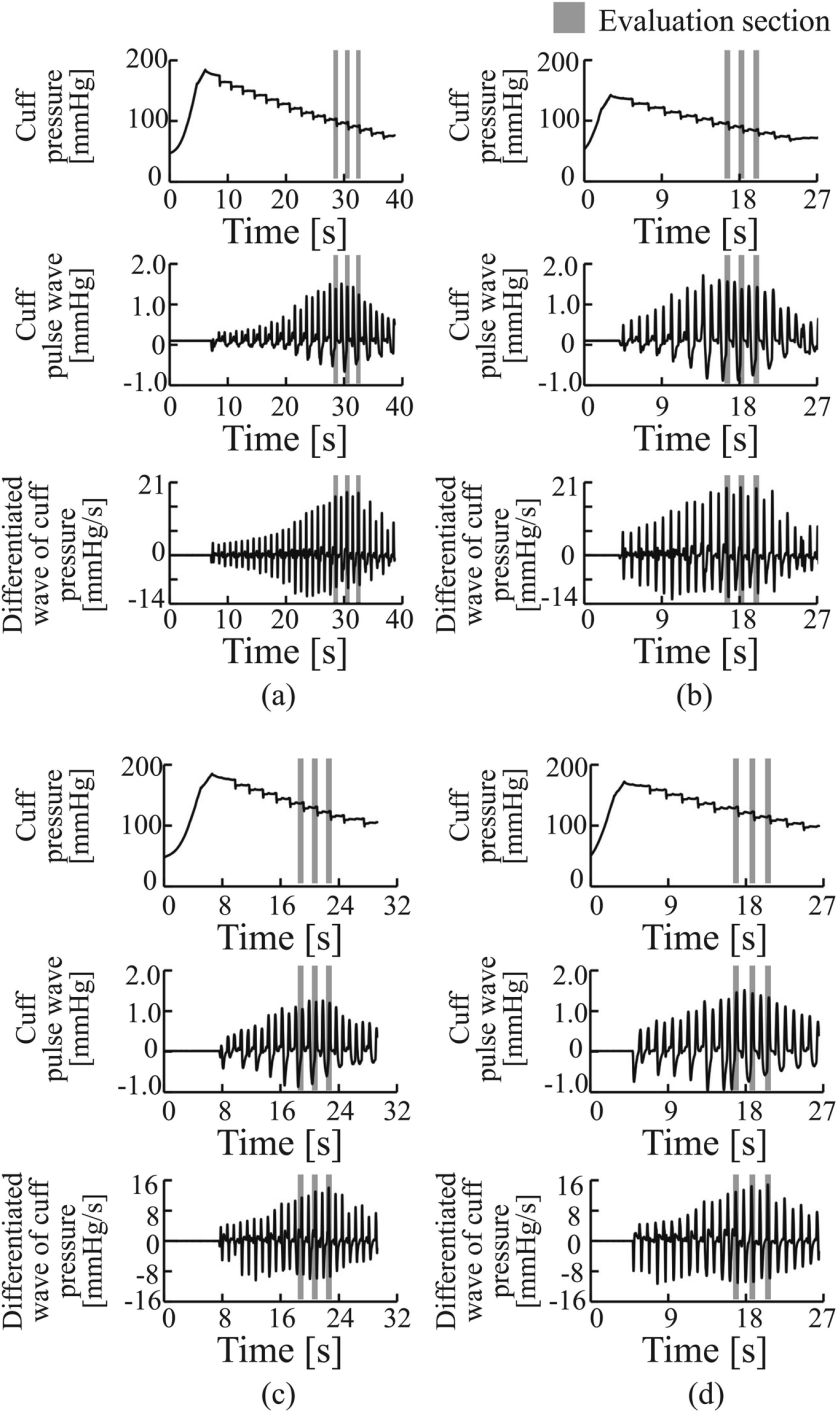
Examples of measured cuff pressure, cuff pressure waves and differentiated waves of cuff pressure: (**a**) pre-cuff occlusion waves from a healthy participant (Sub. 1); (**b**) post-cuff occlusion waves from a healthy participant (Sub. 1); (**c**) pre-cuff occlusion waves from a participant at a high risk of arteriosclerosis (Sub. 30); (**d**) post-cuff occlusion waves from a participant at a high risk of arteriosclerosis (Sub. 30).

Figure 1(a and b) show the examples of the measured waveforms from Sub. 1 (a healthy participant) and Sub. 30 (a participant at a high risk of arteriosclerosis). Figure 1(a and c) show the pre-cuff occlusion cuff pressure, cuff pulse wave and cuff pulse wave velocity waveforms, while Fig. 1(b and d) show the corresponding post-cuff occlusion waveforms. The shaded areas are the data intervals used for calculating the arterial viscosity. The amplitude and velocity of the post-cuff occlusion cuff pulse wave were greater than those of the pre-cuff occlusion cuff pulse wave in both the healthy participants and the participants at a high risk of arteriosclerosis. The pre- and post-cuff occlusion arterial viscosity values for Sub. 1 were 1.71 and 1.72, respectively. The change ratio of arterial viscosity %*η* was 65.4%, as shown in Fig. 1. The results indicated that the third arterial viscosity measured after the cuff occlusion was almost equal to that measured before the cuff occlusion, and the change in the arterial viscosity was a positive value. However, the pre- and post-cuff occlusion arterial viscosity values for Sub. 30 were 5.69 and 2.94, respectively. The change ratio of arterial viscosity %*η* was −57.4%, as shown in Fig. 1. The results indicated that the third arterial viscosity measured after the cuff occlusion was decreased compared with the arterial viscosity measured before the cuff occlusion.

**Figure 2:**
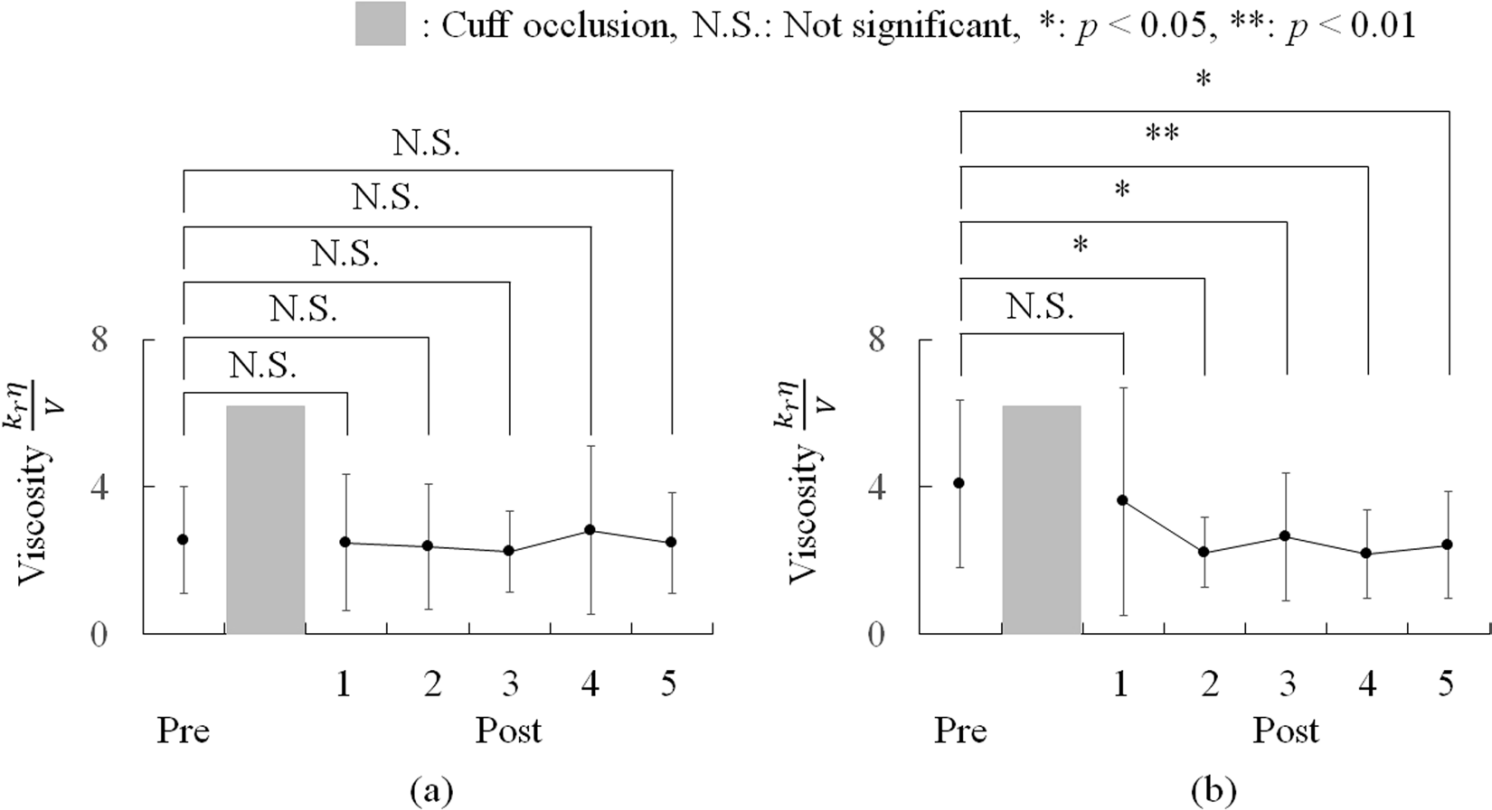
Estimated results of viscosity $\frac{{k}_{r}\eta }{V}$ during the ezFMD test: (**a**) average viscosity $\frac{{k}_{r}\eta }{V}$ of the healthy participants; (**b**) average viscosity of the subjects at a high risk of arteriosclerosis.

Figure 2 shows the transitions of arterial viscosity calculated at each blood pressure measurement. Figure 2(a and b) show the mean values for the healthy participants and the participants at a high risk of arteriosclerosis, respectively, and the shaded area represents the cuff occlusion. Figure 2(a) shows that, during the pre- and post-cuff occlusions, the arterial viscosity values of the healthy subjects are almost constant, and Fig. 2(b) shows that during the post-cuff occlusion, the arterial viscosity values of the subjects at a high risk of arteriosclerosis were lower than the pre-cuff occlusion arterial viscosity values. There was no significant difference in the estimated arterial viscosities between the pre- and post-cuff occlusions in the healthy participants. In contrast, the viscosity of the pre-cuff occlusion was significantly higher than that of the post-occlusions, except that for the first post-cuff occlusion in the participants at a high risk of arteriosclerosis.

**Figure 3:**
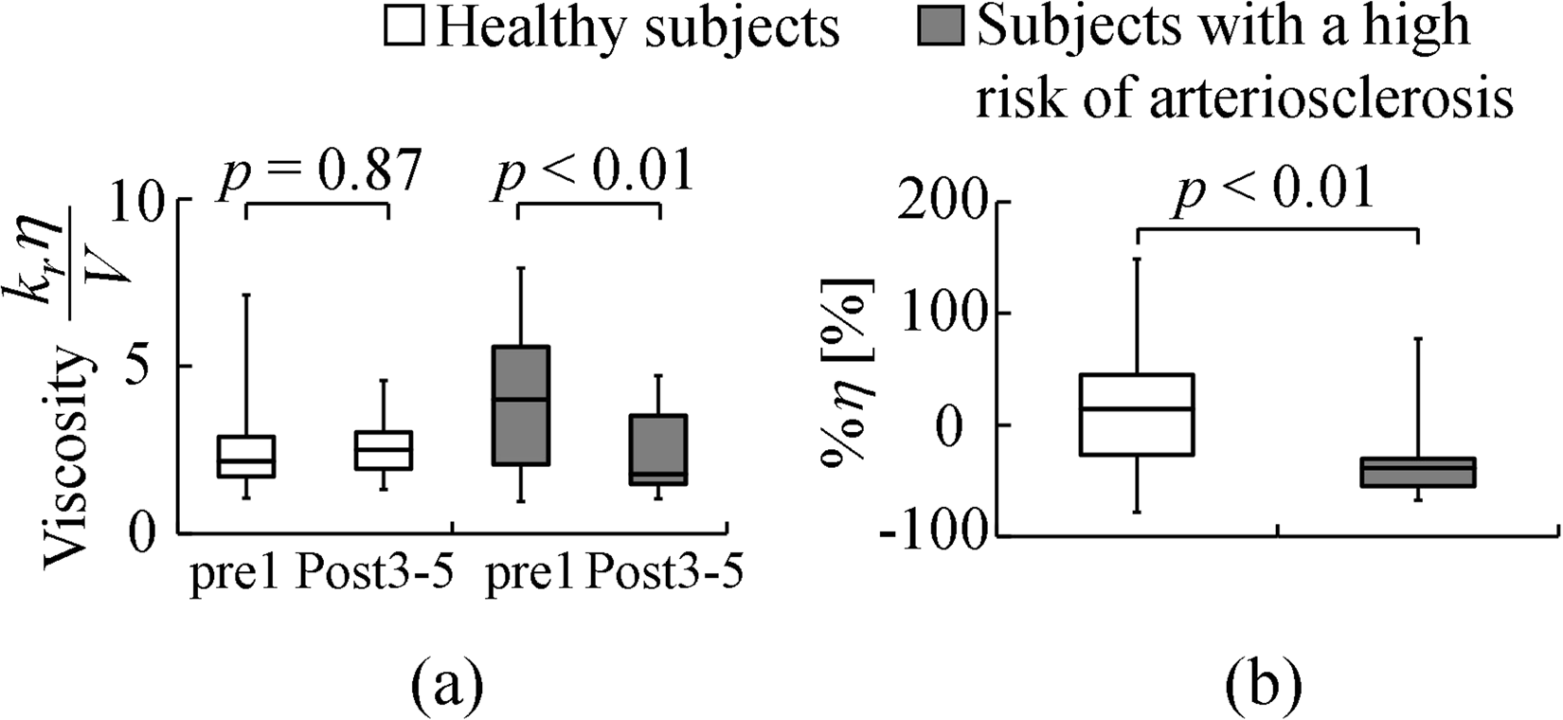
Estimated viscosity of healthy participants and participants at a high risk of arteriosclerosis: (**a**) comparison of pre- and post-cuff occlusions; (**b**) comparison of the change ratio of arterial viscosity %*η*.

Figure 3 shows the comparative results of the pre- and post-cuff occlusion arterial viscosity. Figure 3(a) shows the average arterial viscosity values in the healthy participants and the participants at a high risk of arteriosclerosis. There was no significant difference in the arterial viscosity between pre- and post-cuff occlusion in the healthy participants (pre-cuff occlusion: 2.56 ± 1.45, post-cuff occlusion: 2.51 ± 0.85, *p* = 0.87). However, the post-cuff occlusion arterial viscosity measured in the participants at a high risk of arteriosclerosis was significantly lower than the pre-cuff occlusion arterial viscosity (pre-cuff occlusion: 4.09 ± 2.26, post-cuff occlusion: 2.42 ± 1.28, *p* = 0.0016). Figure 3(b) shows a comparison of the change ratio of arterial viscosity %*η* for the healthy participants and those at a high risk of arteriosclerosis (healthy participants: 17.6 ± 53.8%, participants at a high risk of arteriosclerosis: −29.3 ± 39.5%). The change ratio of arterial viscosity %*η* of the participants at a high risk of arteriosclerosis was significantly lower than that of the healthy participants (*p* = 0.006).

**Figure 4:**
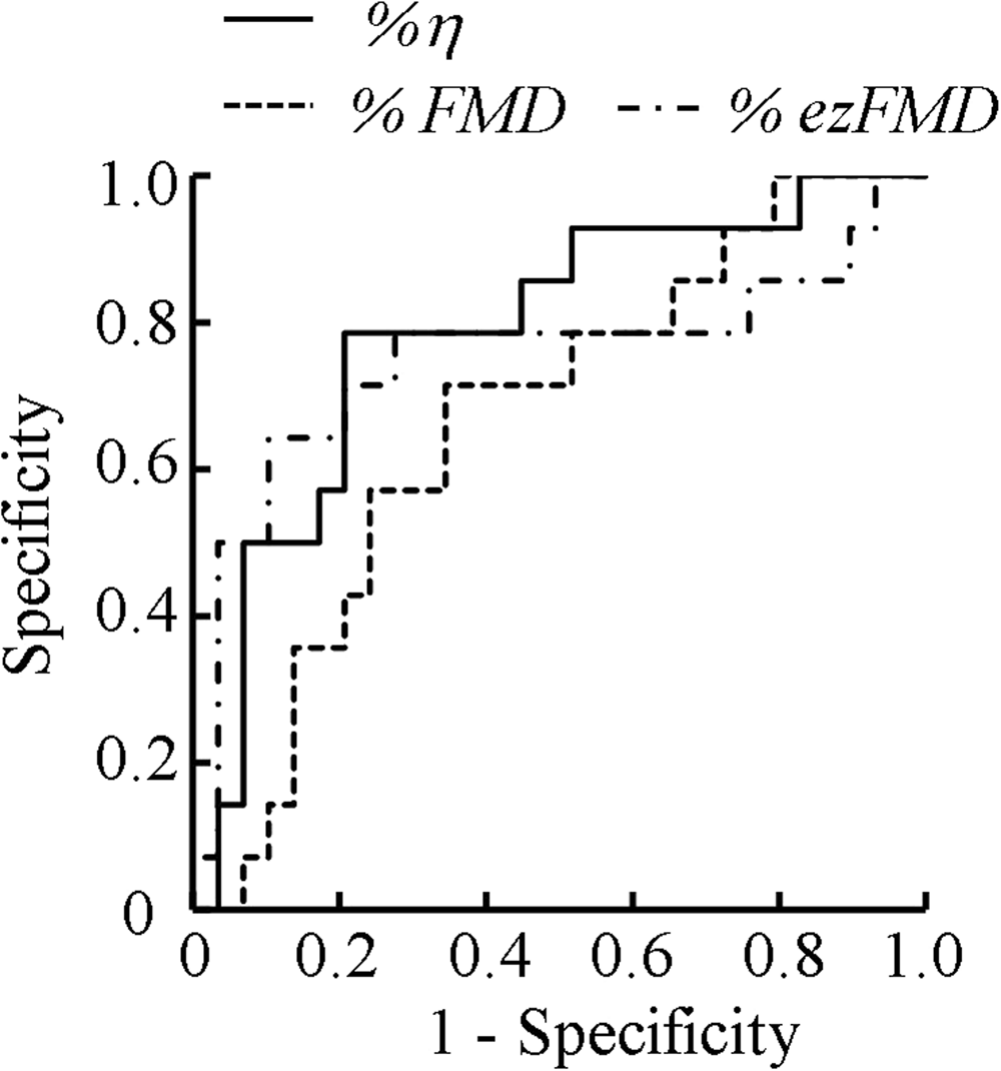
ROC analysis results.

Figure 4 shows the ROC curves of the change ratio of arterial viscosity %*η* and the conventional endothelial function evaluation indices %*FMD* and %*ezFMD*. Table 2 presents the area under the ROC curve (AUC), sensitivity, specificity, and threshold values with the highest ROC analysis discrimination rate. The results showed that the AUC values of the change ratio of arterial viscosity %*η* and the conventional endothelial function evaluation indices %*FMD* and %*ezFMD* were 0.786, 0.695, 0.667, and 0.751, respectively.

**Table 2:** Results of ROC analysis.

	%*η*	%*FMD*	%*ezFMD*
AUC	0.786	0.667	0.751
Sensitivity	0.786	0.714	0.643
Specificity	0.793	0.655	0.897
Threshold	−30.22	6.290	26.54

## Discussion

This paper proposed an estimation method for the vascular viscosity and tested its applicability for endothelial function evaluation. In contrast to the current de facto standard methods such as the FMD and the ezFMD, which evaluate the endothelial function based on changes in vascular diameters, the proposed method uses the vascular viscosity as the arterial mechanical characteristic. The performed ROC analysis showed that the proposed method outperformed the FMD and the ezFMD (see Fig. 4 and Table 2). In addition, the proposed method has advantages in terms of clinical applicability because it employs only an oscillometric automatic sphygmomanometer that is commonly used in clinical practice and does not require highly skilled clinical technologists.

In the experiments, we compared the arterial viscosities between the younger healthy group and the older high-risk group (see Tab. 1) to understand the artery behaviors caused by the cuff occlusion. During post-cuff occlusion, the cuff pulse wave and the cuff pulse wave velocity were increased compared with the corresponding pre-cuff occlusion values for both the healthy participant (Sub. 1) and the participant at a high risk of arteriosclerosis (Sub. 30). This indicates that, even for the participant at a high risk of arteriosclerosis, endothelium-derived NO can be detectable, although diminished^17^. However, the arterial viscosity showed a different trend between the healthy participants and the participants at a high risk of arteriosclerosis. The estimated post-cuff occlusion viscosity did not change significantly compared with the pre-cuff occlusion values in the healthy participants, and the post-cuff occlusion values were decreased compared to the pre-cuff occlusion values for participants at a high risk of arteriosclerosis (Fig. 1).

The transition of the arterial viscosity for each blood pressure measurement also exhibited different trends between the healthy participants and the participants at a high risk of arteriosclerosis. As shown in Fig. 2, the estimated arterial viscosities of the healthy participants remained almost constant from the first post-cuff occlusion, and there were no significant differences in the viscosities between the pre- and post-cuff occlusions. In contrast, in the high-risk participants, significantly low viscosities were found through the second to the last post-cuff occlusion compared to that of the pre-occlusion, but there was no significant difference in the viscosity between the pre-occlusion and the first post-occlusion. This experimental finding in the high-risk participants is consistent with the previous study by Tagawa *et al*.^18^ where they reported delayed NO effects during reactive hyperaemia in human forearm vessels. Specifically, their finding that the role of NO during the early phase of reactive hyperaemia is minimal can explain that fact that the estimated viscosities in this paper did not change significantly in the first post-occlusion. In addition, modest yet significant effects of NO in the mid-to-late phase can explain the difference between the healthy participant group and the high-risk participant group: The effects of NO in the mid-to-late phase in the healthy participant group counteract the significant decreases in the viscosities after the second pre-cuff occlusions in the high-risk participant group. Further, the nearly constant viscosities observed through the first to the last post-occlusion in the healthy participants and through the second to the last post-cuff occlusions in the high-risk participants could be caused by the effects of the post-cuff occlusions. Because the post-cuff occlusions were applied for every 30 s, and they were conducted based on the protocol of the ezFMD^12,13^, the short intervals between the consecutive post-cuff occlusions may impose continuous stimulation to the endothelial function and prevent the viscosities from returning to the normal levels.

**Figure 5:**
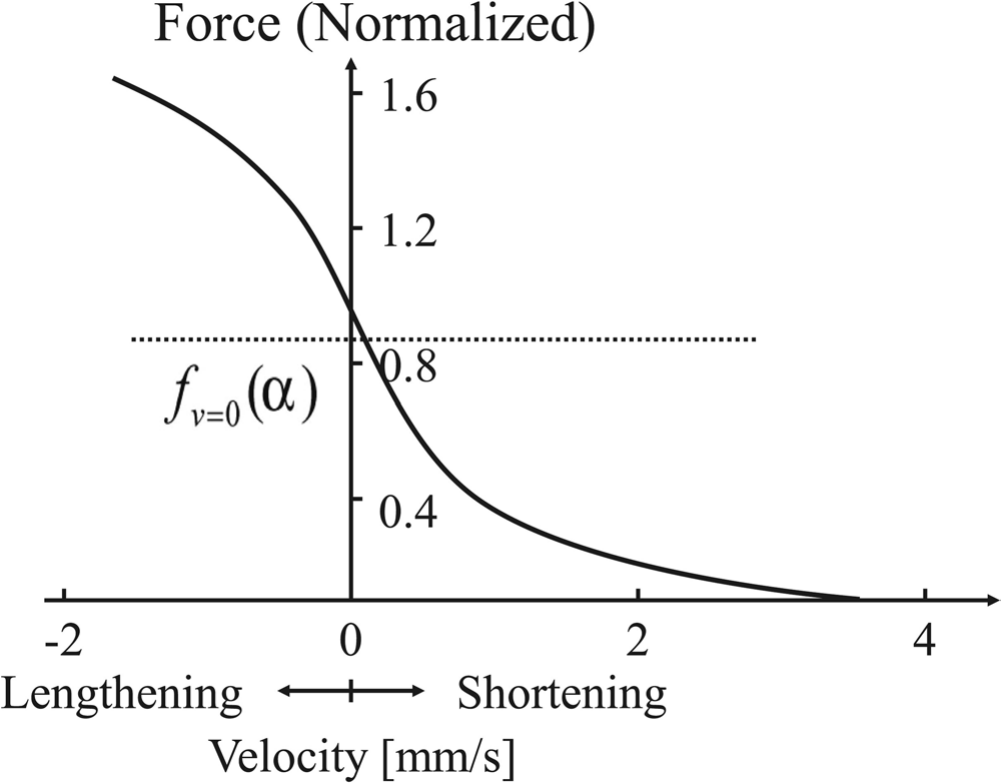
Force-velocity (load-velocity) curves^19^.

**Figure 6:**
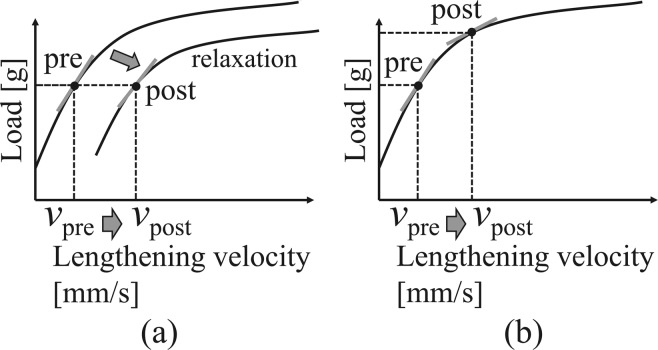
Pre- and post-cuff occlusion changes in the muscle mechanical characteristics and viscosity calculated from the relationships of the slope of tangent: (**a**) healthy participants; (**b**) participants at a high risk of arteriosclerosis.

The difference between the viscosities estimated from the two subject groups could be attributed to the fact that the vascular endothelial function maintains the vascular viscosity, and vascular dysfunction significantly decreases the arterial viscosity after occlusion. However, this straightforward interpretation does not consider the behaviors of the vascular smooth muscle during vasodilation. Mashima *et al*.^19^ reported the relationship between the load exerted on the muscle and the lengthening velocity of the muscle, as shown in Fig. 5; here, the load increases almost linearly in the low lengthening velocity region but gradually saturates in the high lengthening velocity region. The slope of the tangent line on this curve corresponds to the viscosity of the muscle. Then, the viscosity decreases in the high-risk subjects indicate the increases in the lengthening velocities caused by reactive hyperaemia after the pre-cuff occlusion, as shown in Fig. 6(b). In contrast, the viscosities of the healthy subjects remained almost constant even after the pre-cuff occlusion when reactive hyperaemia increased the lengthening velocity. This fact indicates that the relaxation of the vascular smooth muscle, and thus the decrease in the arterial stiffness caused by the endothelial function result in the increase in the vasodilation velocity, and load-lengthening velocity curve of the smooth muscle may be shifted as shown in Fig. 6(a). Therefore, the viscosity changes in the high-risk subject group suggest the impaired ability to change the arterial mechanical properties associated with endothelial dysfunction.

We also confirmed that the change ratio of arterial viscosity %*η* was significantly different between the healthy subjects and those at a high risk of arteriosclerosis; the change ratio of arterial viscosity %*η* in the healthy subjects showed a large variation (Fig. 3(b)). The ability of the change ratio of arterial viscosity %*η* to discriminate the healthy subjects and those at a high risk of arteriosclerosis was greater than or equal to that of %*FMD*^11^, which is a de facto standard index for evaluating vascular endothelial function. These results also indicate that the proposed index %*η* could be used to evaluate vascular endothelial function (see Fig. 4 and Table 2). The major limitation here is that the data presented in this paper could be potentially biased owing to age effects because the healthy group only included younger subjects (21.1 ± 1.85 years) and the high-risk group only included older subjects (63.6 ± 9.24 years). Although we confirmed that the proposed %*η* can differentiate between the healthy and high-risk groups, the age effects have to be examined in future studies by cross-comparison between the high-risk younger and healthy younger groups and between the high-risk older and healthy older groups.

The experimental results confirmed the efficacy of the estimated arterial viscosity in discriminating between the healthy subjects and those at a high risk of arteriosclerosis. This indicates that the estimated arterial viscosity can be used as a risk measure of arteriosclerosis. Further, the proposed method may also be applied to assess the risks of diseases related to arterial viscosity. For example, Lionnet *et al*. reported that hyperviscosity is associated with the pathogenesis of arterial or venous thrombosis, and it can cause the life-threatening complications observed in hemoglobin SC patients such as pulmonary embolism and bone marrow necrosis^20,21^. Further analysis on the relationships between these diseases and the estimated arterial viscosity may expand the application scope of the proposed method.

## Methods

### Subjects

A total of 43 adults (average age ± standard deviation: 34.9 ± 20.9 years) participated in the experiment for measuring the proposed change ratio of arterial viscosity %*η*. The subject breakdown is as follows: 29 healthy subjects (age: 21.1 ± 1.9 years, Subjects 1–29), and 14 subjects at a high risk of arteriosclerosis (age: 63.6 ± 9.2 years, Subjects 30–43). Healthy subjects had no history of cardiovascular disease, liver disease, renal disease, autoimmune disease, or malignancy and had no coronary risk factors, including hypertension, dyslipidaemia, diabetes mellitus, and smoking. Subjects at a high risk of arteriosclerosis were defined as those affected by one or more of the following conditions: hypertension, diabetes, and dyslipidaemia. Hypertension was defined as having a systolic blood pressure of ≧140 mmHg or a diastolic blood pressure of ≧90 mmHg measured in a sitting position on at least three different occasions^22^. Diabetes mellitus was defined according to the guidelines of the American Diabetes Association^23^. Dyslipidaemia was defined according to the third report of the National Cholesterol Education Program^24^.

This experiment was conducted in accordance with the Declaration of Helsinki and was subject to the approval of the Epidemiological Research Ethics Review Committee of Hiroshima University (https://upload.umin.ac.jp. Unique identifier. UMIN000004902). Informed consent was obtained from the subjects.

### Study Protocol

An automatic sphygmomanometer (OPV-1510, Nihon Kohden Corporation; cuff: YP-963T, Nihon Kohden Corporation) widely used in general clinical practice was used to measure the cuff pressure and cuff pulse wave in this experiment. The cuff pressure and cuff pulse wave data were saved on a multimedia card (Transcend) inside the device at a sampling frequency of 125 Hz. The subjects assumed a supine position and were allowed to rest. Their blood pressure was then measured once before the 5-min cuff occlusion and five times after that occlusion. First, to verify the change in arterial viscosity from pre- to post-cuff occlusion, the arterial viscosities *k*_*r*_*η*/*V* were measured in the healthy subjects and the subjects at a high risk of arteriosclerosis. Statistical processing was performed with the Wilcoxon signed rank-sum test, which is a nonparametric test, at a significance level of 5%. Next, the change ratio of arterial viscosity %*η* of the healthy subjects was compared with that of the subjects at a high risk of arteriosclerosis. Statistical processing was performed using the Mann–Whitney U test, which is a nonparametric test, at a significance level of 5%. Furthermore, to examine the accuracy of the change ratio of arterial viscosity %*η* for discriminating the healthy subjects and those at a high risk of arteriosclerosis, the discrimination accuracy of %*η* was compared with that of %*FMD*^11^ and %*ezFMD*^13^, which are also indices for evaluating vascular endothelial function. Comparison of the discrimination accuracies among the indicators was conducted via ROC analysis, which is an accuracy screening method, and comparing the corresponding AUC values.

### Log-linearized under-cuff viscosity index

#### Algorithm for estimating arterial viscosity

Conventionally, it is known that the relationship between the vascular diameter and intravascular pressure is nonlinear in humans^25^. Hayashi *et al*. measured the static arterial diameter *invivo* when artificial internal pressure was applied to the artery. In the physiological pressure range (60 to 160 mmHg), the results showed that the relationship between the logarithmic value of the intravascular pressure for the reference intravascular pressure and the ratio of the vascular diameter for the reference vascular diameter was linear. Thus, Hayashi *et al*. proposed the Stiffness Parameter^25^, which is an estimation index of arterial elasticity and is less susceptible to changes in intravascular pressure. However, it is difficult to express vascular characteristics only using elastic characteristics because *invivo* experiments have reported arteries to be viscoelastic bodies rather than simple elastic bodies^26,27^. To resolve this issue, Hirano *et al*. proposed the log-linearized peripheral arterial viscoelastic model and evaluated the arterial viscosity considering the nonlinear relationship between the peripheral intravascular pressure and peripheral artery diameter^26^. The arterial viscoelastic estimation method proposed by Hirano *et al*. requires the simultaneous measurement of continuous arterial pressures and changes in vascular diameter, which limits the clinical applications of the method. Evaluating the arterial viscosity with an automatic oscillometric sphygmomanometer, which does not require continuous blood pressure measurements, may broaden the clinical applications of this method. This paper proposes an arterial viscoelastic model in which blood pressure is measured by the cuff of an automatic sphygmomanometer wrapped around the upper arm. The proposed model is based on the log-linearized peripheral arterial viscoelastic model proposed by Hirano *et al*. The log-linearized peripheral arterial viscoelastic model is expressed as follows:

1 $$p(t)\cong \tilde{\mu }\ddot{\varepsilon }(t)+\tilde{\eta }\dot{\varepsilon }(t)+\exp \{\tilde{\beta }\varepsilon (t)+{P}_{{\tilde{\beta }}_{0}}+{P}_{{\tilde{\beta }}_{nl}}(\varepsilon (t))\},$$

where *t* represents time, *P*(*t*), ${P}_{{\tilde{\beta }}_{0}}$, and ${P}_{{\tilde{\beta }}_{nl}}(\varepsilon (t))$ are the blood pressure, DC component of the intravascular pressure, and error component based on $\tilde{\beta }\varepsilon (t)$ occurring when the blood pressure drops, respectively. $\tilde{\mu }$, $\tilde{\eta }$, and $\tilde{\beta }$ are coefficients corresponding to the arterial wall inertia, viscosity, and stiffness, respectively. *ε*(*t*), $\dot{\varepsilon }(t)$, and $\ddot{\varepsilon }(t)$ represent the strain of the arterial diameter, strain velocity, and strain acceleration, respectively. Hirano *et al*. measured *ε*(*t*) $\dot{\varepsilon }(t)$ and $\ddot{\varepsilon }(t)$ using a photoplethysmograph. In this study, *ε*(*t*), $\dot{\varepsilon }(t)$, and $\ddot{\varepsilon }(t)$ were determined based on the changes in the vascular volume under the cuff measured by the automatic oscillometric sphygmomanometer. Based on Equation (1), the vascular mechanical properties were approximated with the following equation, using the volumetric vascular strain and volume elasticity^28^:

2 $$p(t)\cong \hat{\mu }\frac{d\ddot{V}(t)}{V}+\hat{\eta }\frac{d\dot{V}(t)}{V}+\exp \{\hat{\beta }\frac{dV(t)}{V}+{p}_{{\hat{\beta }}_{0}}+{p}_{{\hat{\beta }}_{nl}}(\frac{dV(t)}{V})\},$$

where the pressure *p*(*t*) applied from inside the vessel on the outside is considered positive. $\hat{\mu }$, $\hat{\eta }$, and $\hat{\beta }$ are coefficients corresponding to the arterial wall inertia, viscosity, and log-linearized arterial wall stiffness (volume modulus), respectively. Additionally, *dV*(*t*)/*V*, $d\dot{V}(t)/V$, and $d\ddot{V}(t)/V$ are the volumetric vascular change, volumetric change velocity, and volumetric change acceleration, respectively. *dV*(*t*) expresses the change in vascular volume *V* when *p*(*t*) = 0; in an automatic oscillometric sphygmomanometer, slight pressure fluctuations occur in the cuff (cuff volume pulse wave *P*_*pulse*_(*t*)) in response to this change in vascular volume *dV*(*t*).

**Figure 7:**
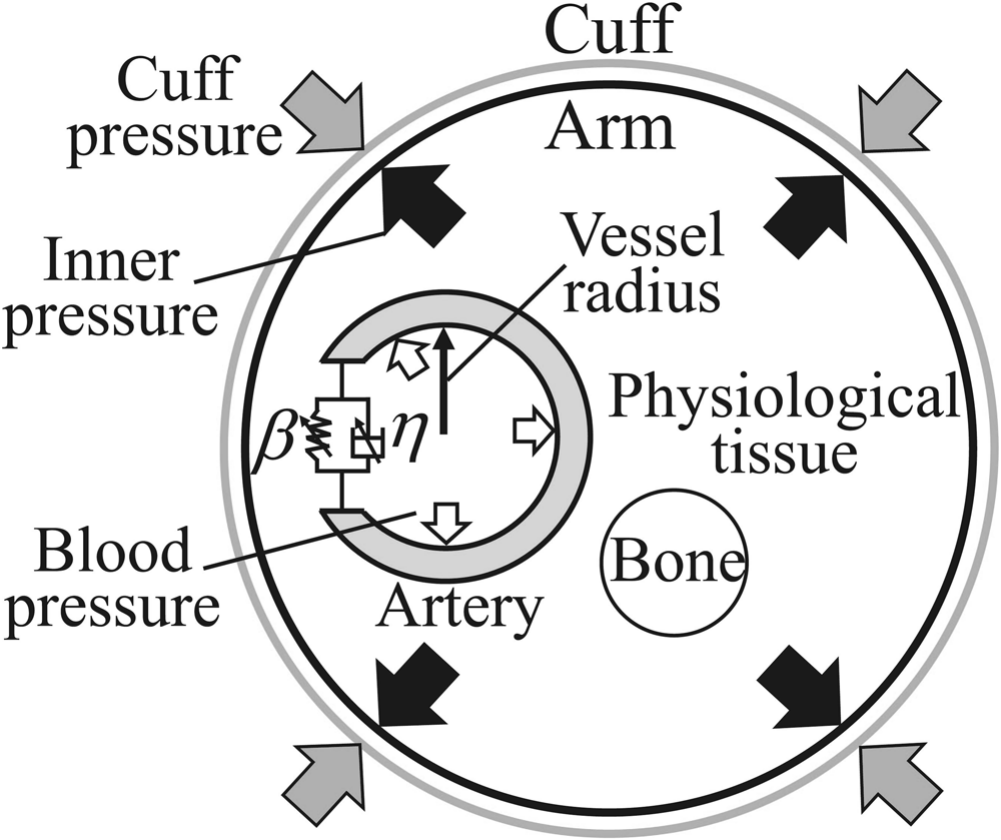
Schematic of an artery in the upper arm surrounded by a cuff.

In this study, viscoelastic parameters were estimated using biological signals obtained from the automatic oscillometric sphygmomanometer and Equation (2). The upper arm covered with the cuff was strongly compressed, and biological tissues other than the artery were assumed to be incompressible while measuring blood pressure^29^. The cuff pressure can be considered to act from the outside toward the inside of the arterial wall, as shown in Fig. 7. The pressure *p*(*t*) acting on the arterial wall is then given as the difference between the intravascular pressure *P*_*b*_(*t*) and cuff pressure *P*_*cuff*_(*t*):

3 $$p(t)={P}_{b}(t)-{P}_{cuff}(t).$$

It was assumed that the internal pressure and volume of the cuff were constant. The Boyle–Charles law holds for the internal pressure, and the cuff volume within the cuff pressure range was used while measuring^30,31^. The volumetric vascular change *dV*(*t*) was then approximated using the following equation because it is proportional to the cuff pulse wave *P*_*pulse*_(*t*)^13^:

4 $$dV(t)={k}_{r}{P}_{pulse}(t),$$

where *k*_*r*_ is a positive constant. Considering only the time at which *dV*(*t*) = 0 and *P*_*b*_(*t*) is equal to or higher than the mean blood pressure, the pressure ${p}_{{\hat{\beta }}_{nl}}(dV(t)/V)$ in Equation (2), which is below the mean blood pressure, can be ignored. Equation (2) can be converted using Equations (3) and (4) when the arterial inertia is ignored owing to its minute value.

5 $${P}_{b}(t)-{P}_{cuff}(t)=\frac{{k}_{r}\hat{\eta }}{V}{\dot{P}}_{pulse}(t)+\exp \{{p}_{{\hat{\beta }}_{0}}\},$$

where *t* is defined as the time at which the cuff pulse wave crosses 0 (*P*_*pulse*_(*t*) = 0). Equation (5) represents the model of the arterial viscosity estimation proposed in this paper. The arterial viscosity is estimated based on this model equation.

In this study, the automatic oscillometric sphygmomanometer reduced the cuff pressure stepwise for every two beats detected. The arterial viscosity was calculated using only the cuff pulse wave of the second beat in the two beats detected by decompression because every first beat could potentially be affected by disturbances caused by cuff decompression. The commercial automatic oscillometric sphygmomanometer displays the systolic, diastolic, and mean arterial pressure values for each arterial pressure measurement. The mean arterial pressure is determined based on the maximum peak-to-peak value of the cuff pulse wave, which indicates the minimum difference between the intravascular and extravascular pressures. Systolic and diastolic arterial pressures are determined by an algorithm in each automatic sphygmomanometer, which satisfies a certain criterion. The algorithm for calculating systolic and diastolic pressures is not based on the physical properties of arterial vessels^29^. Thus, the arterial viscosity obtained using the proposed method was estimated using the cuff pressure around the mean arterial pressure and the cuff pulse wave velocity at which the cuff pulse wave amplitude was maximal.

The time *t* satisfying Equation (5) and the following condition is defined as the reference time t_*_. This condition is such that the maximum pulse wave and maximum pulse wave velocity observed from the three beats, i.e., the maximum pulse wave and adjacent waves of its maximum wave, are obtained. At the time *t* satisfying Equation (5), the time before *j* beats ($j\in {\boldsymbol{{\mathbb{N}}}}$) was set to *t*_*j*_. The difference between the time t_*_ and the time *t*_*j*_ was then obtained from Equation (5):

6 $$({P}_{cuff}({t}_{\ast })-{P}_{cuff}({t}_{j}))=\frac{{k}_{r}\hat{\eta }}{V}({\dot{P}}_{pulse}({t}_{j})-{\dot{P}}_{pulse}({t}_{\ast })),$$

where *P*_*b*_(t_*_) is assumed to be equal to *P*_*b*_(*t*_*j*_). In this study, Equation (6) was simultaneously solved for all times *t*_*j*_. The solution ${k}_{r}\hat{\eta }/V$ obtained using the linear least squares method is defined as the estimated arterial viscosity value.

#### Evaluation of vascular endothelial function

In humans, the peripheral part of the upper arm becomes oxygen deficient with the cuff occlusion of the upper arm. The blood flow increases immediately after release of the cuff occlusion because the state of oxygen deficiency in the periphery needs to be eliminated. In case of normal vascular endothelial function is, NO is released from the endothelial cells because the shear stress associated with the increase in blood flow is applied to the vascular wall, causing the vascular smooth muscle to relax and the pulsation corresponding to the vascular volume to increase^29^. However, it becomes difficult to release NO from endothelial cells when the vascular endothelial function is abnormal, which prevents vascular smooth muscle relaxation^17^. By applying this biological reaction, vascular endothelial function was evaluated based on the arterial viscosity calculated before and after cuff occlusion.

**Figure 8:**
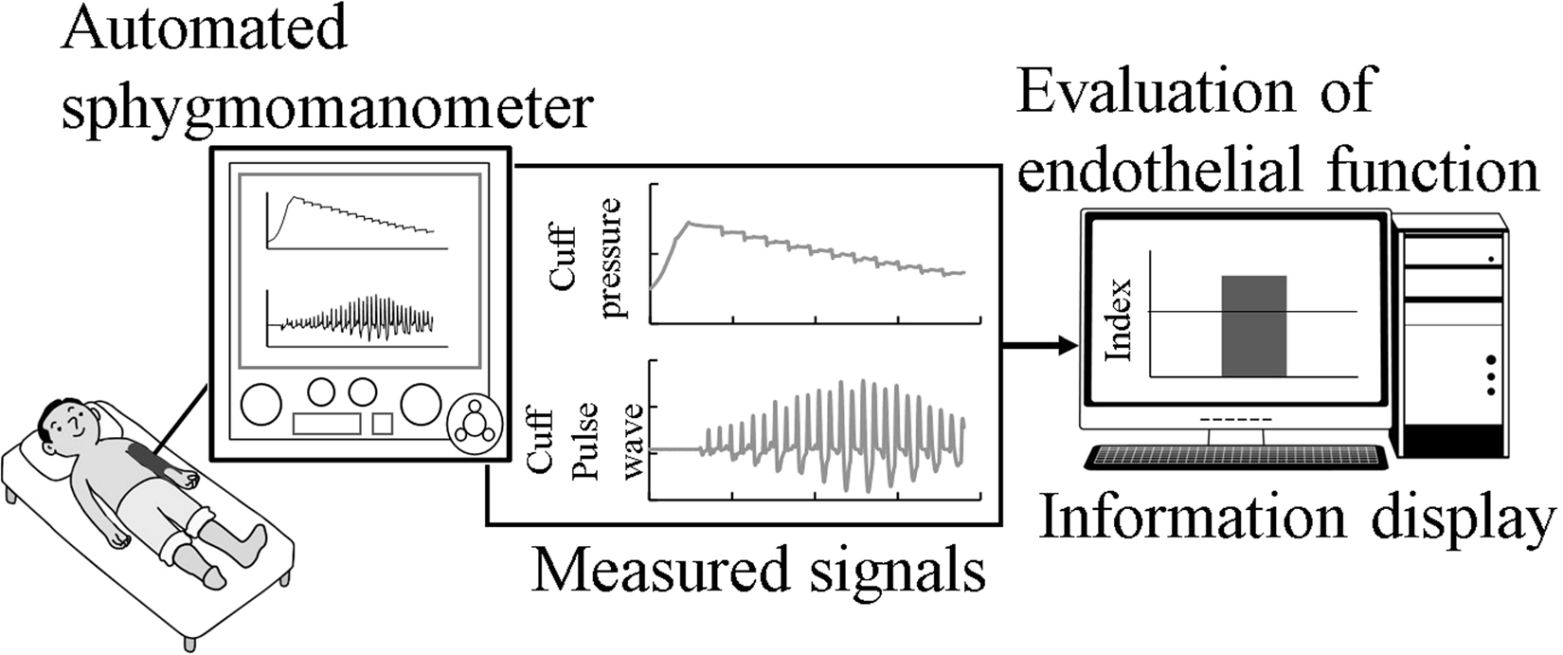
Overview of the proposed examination system.

A schematic of the proposed vascular endothelial function evaluation system is shown in Fig. 8. The arterial viscosity of the proposed model is calculated from the cuff pressure and cuff pulse wave measured by the automatic oscillometric sphygmomanometer. The evaluated vascular endothelial function is displayed on the monitor. The protocol of the ezFMD test^13^ was adopted as the experimental protocol. The ezFMD test is a method for evaluating vascular endothelial function, which is calculated from the change ratio of the pulse wave amplitude before and after cuff occlusion. The ezFMD test does not require an ultrasonic device for evaluating the endothelial function. The subject assumes a supine position and rests during the test. The subject’s arterial pressure is measured once before cuff occlusion and five times after cuff occlusion. The period of cuff occlusion is 5 min. The cuff pressure and cuff pulse wave during the ezFMD test are stored as numerical data. The change rate of arterial viscosity %*η* from pre- to post-cuff occlusion is expressed as follows:

7 $${\rm{ \% }}\eta =(\frac{{\frac{{k}_{r}\eta }{V}}_{{\rm{p}}{\rm{o}}{\rm{s}}{\rm{t}}3-5}-{\frac{{k}_{r}\eta }{V}}_{{\rm{p}}{\rm{r}}{\rm{e}}}}{{\frac{{k}_{r}\eta }{V}}_{{\rm{p}}{\rm{r}}{\rm{e}}}})\times 100,$$

where (*k*_*r*_*η*/*V*)_post3−5_ is the average arterial viscosity value calculated from the 3rd to 5th post-cuff occlusion arterial pressure measurement, and (*k*_*r*_*η*/*V*)_pre_ is the pre-cuff occlusion arterial viscosity. The post-cuff arterial viscosity is taken as the average of the 3rd to the 5th measurements to avoid a condition in which the blood flow immediately after the cuff occlusion is unstable. In this study, the proposed method was used to distinguish between healthy subjects and subjects at a high risk of arteriosclerosis, and the vascular endothelial function of the subjects was evaluated using the change ratio of arterial viscosity %*η*.
